# Femtomagnetism in graphene induced by core level excitation of organic adsorbates

**DOI:** 10.1038/srep24603

**Published:** 2016-04-19

**Authors:** Abhilash Ravikumar, Anu Baby, He Lin, Gian Paolo Brivio, Guido Fratesi

**Affiliations:** 1 Dipartimento di Scienza dei Materiali, Università di Milano-Bicocca, Via Cozzi 55 - 20125 Milano, Italia; 2 Dipartimento di Fisica, Università degli Studi di Milano, Via Celoria, 16 - 20133 Milano, Italia

## Abstract

We predict the induction or suppression of magnetism in the valence shell of physisorbed and chemisorbed organic molecules on graphene occurring on the femtosecond time scale as a result of core level excitations. For physisorbed molecules, where the interaction with graphene is dominated by van der Waals forces and the system is non-magnetic in the ground state, numerical simulations based on density functional theory show that the valence electrons relax towards a spin polarized configuration upon excitation of a core-level electron. The magnetism depends on efficient electron transfer from graphene on the femtosecond time scale. On the other hand, when graphene is covalently functionalized, the system is magnetic in the ground state showing two spin dependent mid gap states localized around the adsorption site. At variance with the physisorbed case upon core-level excitation, the LUMO of the molecule and the mid gap states of graphene hybridize and the relaxed valence shell is not magnetic anymore.

Graphene is a two dimensional crystalline allotrope of carbon with honeycomb lattice structure and has been extensively studied since its discovery in 2004^1^,^2^,^3^,^4^. Free standing graphene has a unique electronic band structure where the valence and conduction bands touch at *K* points of the first Brillouin zone forming the Dirac cones^5^. The linear dispersion and the absence of band gap makes graphene a semi-metal with several interesting properties such as ballistic transport^6^, long mean free path at room temperature^7^, high charge carrier mobility with massless relativistic carriers called Dirac Fermions^8^ and anomalous integral and half integral quantum Hall effect^9^,^10^,^11^. These properties suggest graphene and its composites as ideal candidates for efficient gas sensors^12^,^13^, novel spintronic devices^14^ and transparent electrodes for photovoltaics^15^,^16^.

Pristine graphene does not display intrinsic magnetism and this limits its applications in magneto-electronics and active spintronics. This is due to the bipartite lattice structure of graphene explained by Lieb’s theorem^17^. Several methods have been proposed to induce magnetism in graphene such as creating structural vacancies or surface defects^18^,^19^,^20^,^21^,^22^ and polarized edge states^23^,^24^,^25^. But these methods destroy the crystalline integrity of graphene and proves experimentally challenging to maintain the induced magnetism during device fabrication^26^. For these reasons recent studies about partial hydrogenation of graphene^27^,^28^, covalent adsorption of organic molecules^29^,^30^,^31^ and more recently graphene nanoridges with oriented fluorine chains^32^ have attracted a lot of attention. Covalent adsorption of aryl radicals forming periodic superlattices also demonstrated the possibility to achieve long range magnetic order under a magnetic field^33^.

Time dependent effects in magnetism are an important topic of current interest. Ultrafast magnetization (so called femtomagnetism) has been investigated first by Beaurepaire *et al*., who showed that a rapid demagnetization is induced in Ni by using femtosecond optical pulses^34^. In ferromagnetic thin films by using near-infrared femtosecond laser pulses, it is possible to disentangle the spin and orbital components of the magnetic moment and to probe this by femtosecond resolved X-ray magnetic circular dichroism (XMCD)^35^. Very recently, XMCD has allowed for direct measurement of transient magnetic moments in a non-magnet (Cu) caused by injection of spin polarized current from an adjacent ferromagnet (Co)^36^ and transients for spin-resolved currents for graphene coupled to magnetic Co occurring within few femtoseconds have been predicted^37^. Core-level excitations in electron energy loss spectroscopy (EELS) are also at the basis of a study of the magnetic moment in graphene induced by transition metal dopants located at single vacancy, double vacancy and edge defect sites^38^. The core hole clock method^39^ allows one to determine electron transfer times from adsorbed molecules on graphene (with applications including photovoltaics and light emitting diodes^40^,^41^,^42^,^43^,^44^) and has demonstrated that such times can be shorter than the one of core-level de-excitation^45^. While a proper description of the transient properties of the system under study would be necessary to exploit the experiments fully, the core hole clock method is also capable of obtaining spin resolved measurements either by creating resonances with selected spins by circularly polarized light or by spin-resolved detection of emitted electrons^46^,^47^.

The motivation for this work is to understand the effect of core level excitations of adsorbed molecules on graphene, aiming at the limit where only one molecule in a diluted array of adsorbed species is core-excited. We take into account three molecules namely pyridine (C_5_H_5_N), 4-picoline radical (C_6_H_6_N) and pyridine radical (C_5_H_4_N), which are prototypes of different couplings with graphene. Interaction of pyridine with graphene is dominated by van der Waals forces, that of pyridine radical is mainly via chemisorption, while 4-Picoline radical is affected by both van der Waals forces and covalent bonding and represents an intermediate case between pyridine and pyridine radical. We evaluate the electronic properties for the molecules where the N atom is ionized and compare them to the ground state ones. We investigate how the electrons of the system relax around the core hole, inducing or suppressing magnetism in the valence shell depending on the type of adsorption.

In the following section we outline the computational methodology. Then, the results of our models will be presented and discussed in detail followed finally by the conclusions.

## Simulation Details

We perform first principles calculations based on density functional theory (DFT)^48^,^49^,^50^ within the generalized gradient approximation (GGA) framework using Perdew-Burke-Ernzerholf (PBE) exchange correlational functional^51^,^52^ and a plane wave basis set as implemented in the Quantum Espresso platform^53^. The van der Waals interactions make an important contribution to the adsorption of organic molecules and Grimme correction^54^ is included to explain this accurately. The chosen 5 × 7 surface periodicity of the graphene substrate ensures low adsorbate concentration of 6.69 × 10^−3^ Å^−2^ and negligible inter-molecular interactions. A vacuum region of 15 Å is set to minimize the interaction between graphene layers along the z-axis orthogonal to graphene. The plane wave kinetic energy cut-off is 42 Ry and the convergence on the energy and force are 10^−4^ a.u. and 10^−3^ a.u. respectively. The Brillouin zone is sampled using a 3 × 2 × 1 Γ-centered k-point grid for system relaxation and total energy calculations. A 18 × 12 × 1 Γ-centered k-point grid is chosen to calculate the density of states. The simulation settings are tested for free standing graphene and its lattice constant was found to be 2.462 Å, which is in very good agreement with the experimental value of 2.46 Å^55^.

For the excited state calculations, we use a pseudo-potential with a full core hole (FCH) created at the N 1s orbital of the molecule. We always consider a globally neutral system, where an additional electron is given to the valence shell^56^,^57^. Physically, this corresponds to the X-ray absorption at the edge or to the final state of a photoemission experiment assuming that the valence shell relaxes fully around the core hole. In the latter case, a further electron is attracted to the molecular region from the surrounding graphene. This cannot occur in the calculation since the neighboring cells are also excited due to periodic boundary conditions. Hence the necessity to add an electron to the valence shell to make the most representative model of the physical case, where very few molecules are excited simultaneously. This also provides a more effective convergence with respect to unit cell size as thoroughly demonstrated recently^57^.

## Results and Discussion

**Figure 1:**
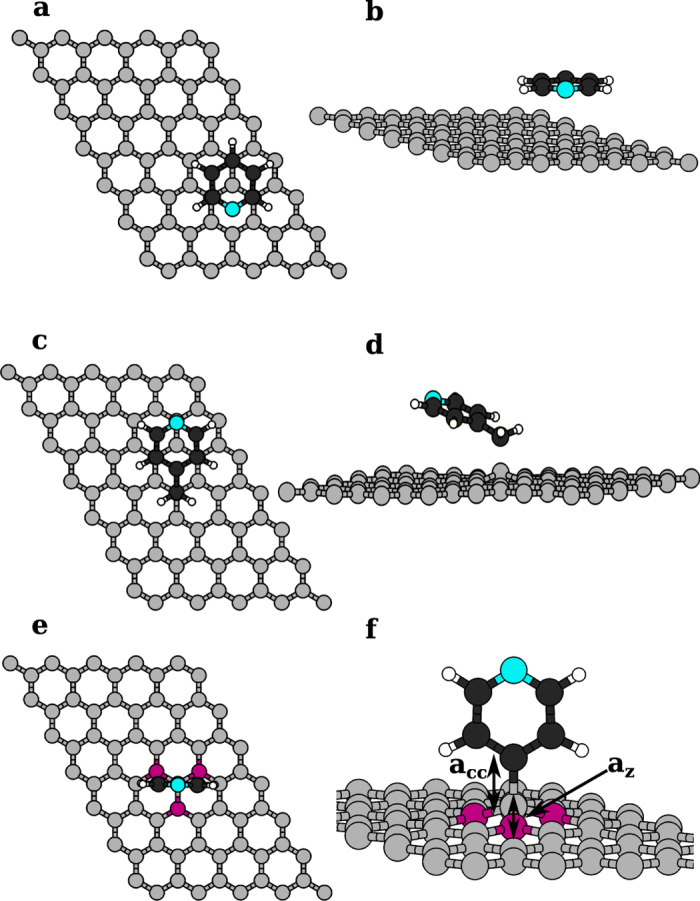
The minimum energy configurations of organic molecules from the top and perspective view for (**a**,**b**) pyridine (**c**,**d**) 4-picoline radical and (**e**,**f**) pyridine radical adsorbed on graphene. The yellow and red spheres represent C atoms of graphene and of the molecule, respectively. The blue spheres stand for N and smaller black ones for H atoms. The perspective view shown in (**f**) highlights the localized distortion in the graphene lattice when it covalently bonds with pyridine radical. There, *a*_*cc*_ represents the molecule-graphene bond length and *a*_*Z*_ is the displacement of the carbon atom of graphene covalently bonded to the radical, as measured with respect to its nearest neighbours.

We start by investigating the most stable configurations of pyridine, 4-picoline radical and pyridine radical adsorbed on graphene taking into account the translational as well as rotational degrees of freedom of the molecule. To check the stability of the system and quantitatively understand the energies involved during the adsorption process, we define the adsorption energy as 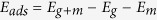. Here 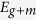 is the total energy of the optimized molecule-graphene system, *E*_*g*_ and *E*_*m*_ are the total energies of the isolated graphene substrate and of the gas phase molecule/radical, respectively. The adsorption energies are summarized in Table 1, along with most relevant structural parameters. We find that pyridine placed parallel to graphene, with its N atom at the center of a graphene ring, is the most stable configuration at low molecular concentration (Fig. 1(a,b)) and its interaction with the substrate is dominated by van der Waals forces. These results are supported by various experimental and theoretical studies of closed shell organic molecules adsorbed on graphene^58^,^59^,^60^. Our most stable configuration displays the pyridine ring symmetrically located with its nitrogen atom at the center of the graphene ring and oriented similar to AB stacking found in graphite. Another arrangement on graphene in which the nitrogen atom is above one of the carbon atoms of graphene has similar adsorption energy, just 6 meV less stable. A small energy difference between these configurations^60^ points to high molecular diffusivity. If we neglect the van der Waals interactions, the adsorption strength is underestimated by one order of magnitude while the adsorption bond length is seriously overestimated. We next consider the 4-picoline (4-Methlypyridine) radical (where we have removed one of the hydrogen atoms of the methyl group) which forms a covalent bond with the free *p*_*z*_ orbitals of graphene as shown in Fig. 1(c,d). For this system, van der Waals forces also play an important role to access the minimum energy configuration since the pyridine *π* cloud remains almost parallel to graphene, at an angle of ~14° and at an average distance of 3.16 Å with respect to the substrate plane. Finally the pyridine radical, when adsorbed on graphene, forms a mostly covalent bond and orients itself perpendicular to the graphene plane as seen in Fig. 1(e). Figure 1(f) shows the perspective view of this configuration. A localized deformation of the graphene lattice due to covalent interaction with the molecule lifts the carbon atom of graphene^61^ by a quantity *a*_*Z*_, defined as the height difference of the bonding carbon atom of graphene with respect to its nearest neighbours. This is visualized clearly in the Fig. 1(f) along with the graphene-molecule bond length (*a*_*cc*_). The results for the adsorption energies are better understood by taking pyridine radical as a reference. Picoline radical, in comparison, shows a lower value of *a*_*cc*_ and *a*_*Z*_, consistently with its lower reactivity. The more negative computed value of *E*_*ads*_ is due to a stronger vdW interaction of its almost planar *π* cloud. In comparing pyridine molecule vs radical, we remark that in the case of covalently bonded systems on graphene, the deformation of the substrate requires a large energy cost that is lowering significantly the adsorption energy: almost 1 eV in similar systems^62^. Our adsorption energy value for pyridine radical is comparable to that of other radicals published therein. On the contrary, vdW-bonded pyridine does not suffer this deformation cost and is stabilized by a larger dispersion interaction, hence the overall larger value of *E*_*ads*_. Our effort will be to understand the effect of chemisorption and physisorption on the system magnetism in the ground and core excited states.

To understand the electronic and magnetic properties of the system, we plot the total density of states (DOS) and that projected onto the molecular orbitals (MOPDOS). The latter is evaluated as^56^,


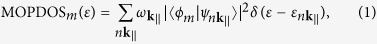


where 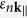 and 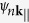 are the Kohn-Sham eigenvalues and eigenvectors, respectively, and 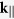 samples the surface Brillouin zone with weights 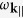, and we represent the Dirac *δ* function by a Gaussian function. The molecular states 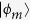 have been evaluated for isolated species taking spin-compensated solutions for the ground state or core-excited one, depending on the case of interest. The dangling bonds of the radicals were saturated with H atoms. The overlaps between wave functions, 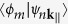, are eventually computed via an atomic basis representation. We refer to the Supplemental Information for more detail.

**Figure 2:**
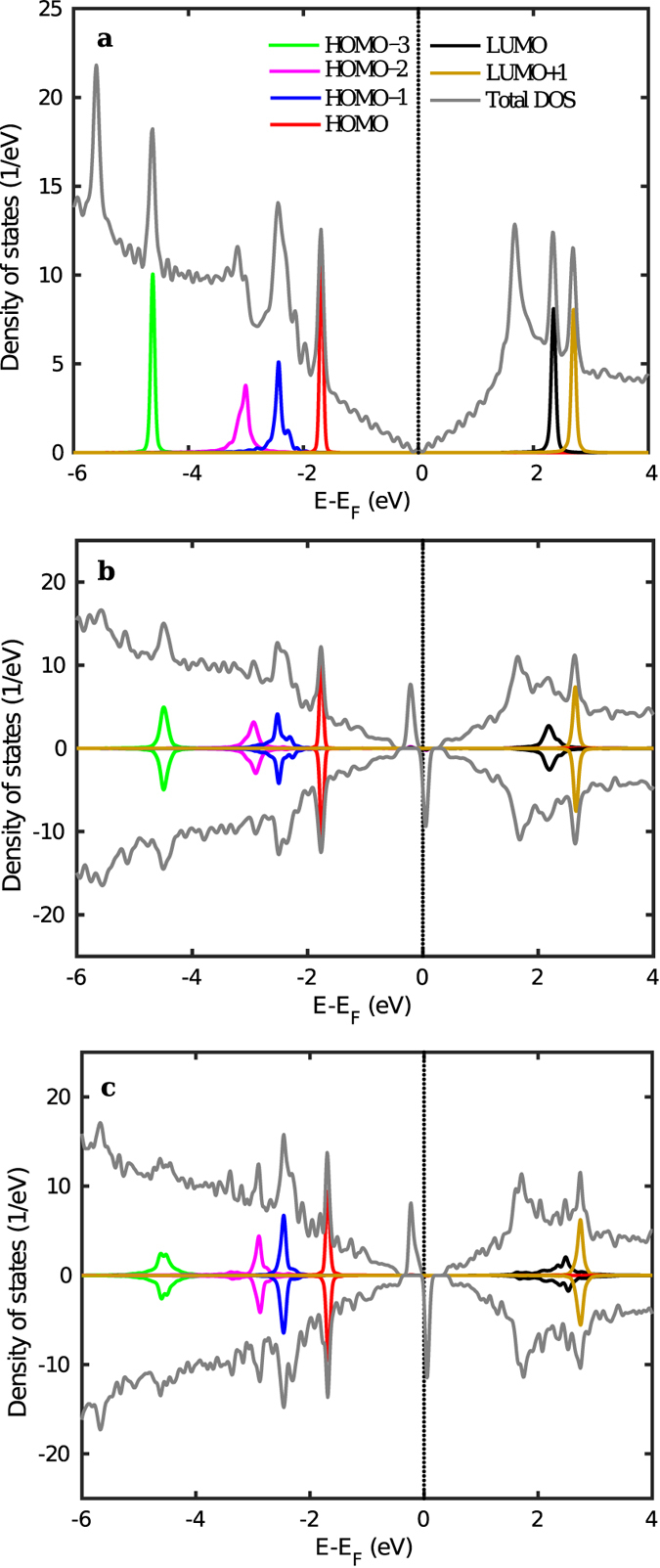
Ground state DOS and DOS projected onto the molecular orbitals for the (**a**) pyridine on graphene, (**b**) picoline radical on graphene and (**c**) pyridine radical on graphene are shown. Values are per spin channel and the spin-minority DOS and MOPDOS are reported with a negative sign in (**b**,**c**) panels. The plot was done with a Gaussian broadening (full width at half maximum) of 0.14 eV in Eq. 1.

In pyridine on graphene configuration as seen in Fig. 2(a), since the molecule is physisorbed and the interaction is dominated by van der Waals forces, we do not see any significant change in the DOS when compared to that of free standing graphene but for the addition of states contributed by the molecule. The Dirac point of graphene remains at the Fermi level and is not significantly perturbed. Since pyridine is a closed shell system with even number of electrons in the valence band, the system remains non-magnetic upon molecular adsorption. Projection of the molecular orbitals of the total system onto the molecular orbitals of pyridine reveals the position of the resonant energy levels of pyridine. The Kohn-Sham HOMO-LUMO band gap of pyridine amounts to about 4 eV and this is supported by other studies using DFT^60^.

Before discussing the adsorption of 4-picoline and pyridine radicals on graphene, we recall that covalent adsorption of species bound to a C atom of graphene induces magnetism in the ground state of the system, as the respective p_*z*_ orbital is taken out of the *π* electron network^28^,^61^,^62^. This consequently opens a band gap whose value depends on the size of the unit cell used in the calculation. Two localized spin-dependent mid gap states at energies above and below the Fermi level appear and the system gains 1 *μ*B magnetic moment. The molecule does not contribute directly to the formation of such states and magnetism is localized around the bonding site on graphene but on the other sublattice^62^. Consequently in the results shown in Fig. 2(b,c) for 4-picoline and pyridine radicals, respectively, the MOPDOS’s reveal that the molecule does not contribute directly to the formation of mid gap states. The main effect on the orbitals of the now-saturated molecule is coupling with the states of the substrate and consequent state-dependent broadening. It is important to realize that different magnetic configurations could be obtained at larger molecular coverage. In particular, adsorption of a second molecule nearby is preferred at the other graphene sublattice and would result into a non-magnetic solution. Adsorption on the same sublattice would lead to ferromagnetic coupling between the two species, but is energetically unfavorable. At large distances between adsorbates, local magnetism would eventually be preserved^61^. Whenever periodic superlattices of covalently bonded species could be formed, long range magnetic order under a magnetic field may be found^33^.

**Figure 3:**
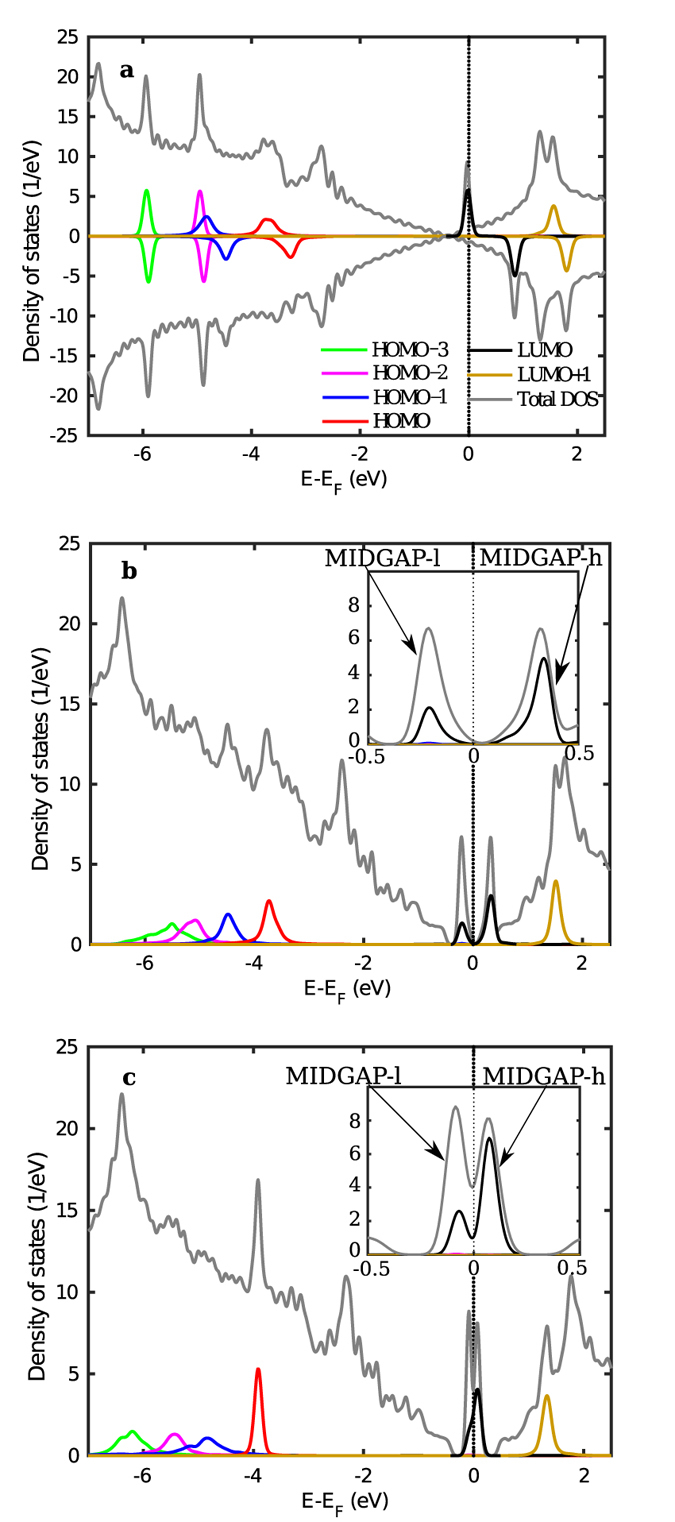
Same as Fig. 2, upon N 1s core-level excitation, for (**a**) pyridine on graphene, (**b**) picoline radical on graphene and (**c**) pyridine radical on graphene are shown. Values are per spin channel and the spin-minority DOS and MOPDOS are reported with a negative sign in panel (**a**). The plot was done with a Gaussian broadening (full width at half maximum) of 0.14 eV in Eq. 1, and of 0.07 eV in the insets which enlarge the region around the Fermi level in panels (**b**,**c**).

We are now in the position to study the electronic and magnetic properties for the three systems when a hole in the N 1s orbital of the molecule is created and one electron is added to the valence shell, as discussed in simulation details. The total DOS and the MOPDOS of the three molecules are shown in Fig. 3. The attractive potential (created by the core hole) results in a shift of the molecular orbitals to lower energies. In the case of pyridine, as seen in Fig. 3(a), one can observe that the Dirac point of graphene in the region of the excited molecule also shifts by about 0.4 eV to accommodate a fraction of the screening charge. The additional valence electron is mostly located in the LUMO of the molecule just below the Fermi level creating a spin polarized configuration with 1 *μ*B magnetic moment localized on the molecule. We remark that this magnetic configuration would occur upon 1s→LUMO excitation or, in case of photoionization, after the time needed to transfer an electron from graphene to the molecule to screen the core hole. As demonstrated by recent experiments for bipyridine molecules on graphene^45^, such electron transfer can occur within the life time of the core excitation that, for N and C K-shells, is of few femtoseconds^63^. After the core hole de-excites, the evolution of the valence system would depend on the specific final state of the de-excitation and the LUMO would possibly shift to higher energy with electron transfer back to graphene.

**Figure 4:**
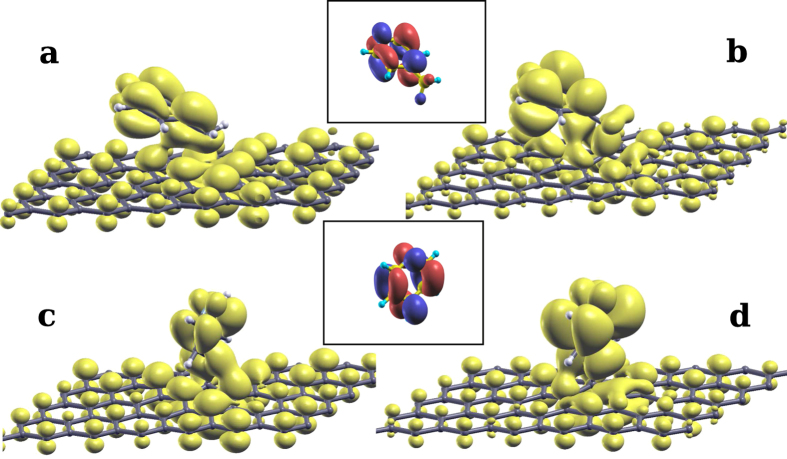
Panels (**a**,**b**) plot the MIDGAP-l and MIDGAP-h states, respectively, formed by hybridizing the mid gap state of graphene with the LUMO of core-excited 4-picoline radical, and showing mostly bonding and anti-bonding amplitude contours, respectively. Panels (**c**,**d**) show the same states for a core-excited pyridine radical. Gas phase LUMO for 4-picoline and pyridine are plotted in the insets.

In the case of covalently bonded 4-picoline radical and pyridine radical, the system is magnetic in the ground state. Upon photo-ionization and relaxation of the valence shell around the core hole, the additional valence electron joins the one formerly in the midgap state and the system valence becomes spin degenerate. It remains so until the excited hole decays with a lifetime of a few femtoseconds. It is seen for the picoline and pyridine radical cases in Fig. 3(b,c) respectively that the LUMO of the molecule shifts down to the Fermi level. There, it hybridizes with the mid gap states of graphene, forming two mixed states at lower and higher energy that we name MIDGAP-l and MIDGAP-h, respectively. The spatial charge distribution at the MIDGAP-l and MIDGAP-h states is evaluated by the energy-integrated local density of states (ILDOS) and is defined as:


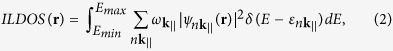


where the energy range (*E*_*min*_ to *E*_*max*_) is chosen to include either the MIDGAP-l or MIDGAP-h states. The ILDOS of the occupied MIDGAP-l and the unoccupied MIDGAP-h are plotted in Fig. 4(a,b), respectively, for the picoline radical, and in Fig. 4(c,d) for the pyridine radical. The LUMO for gas phase 4-picoline and pyridine molecules are also displayed as insets, confirming that the molecular orbital contributing to these states is indeed the LUMO, consistently with the projections seen in Fig. 3(b,c). By examining the relative heights of the peaks in Fig. 3(b,c) we see that the MIDGAP-l state is derived mainly from the mid gap states of graphene, with smaller weight on the molecule. On the contrary, a lower graphene contribution is displayed on MIDGAP-h which is mainly made up of the LUMO of the molecule. Additionally, since the pyridine ring is almost parallel to graphene for 4-picoline radical, its LUMO with *π* symmetry couples strongly with the facing p_*z*_ orbitals of graphene which constitute the midgap states. This results in a larger energy splitting between the hybrid MIDGAP-l and MIDGAP-h states (0.55 eV) seen in the MOPDOS of Fig. 3(b), which is indicative of efficient electron transfer between LUMO and midgap states^64^. Conversely, for the pyridine radical adsorbed normal to graphene, coupling with the midgap state is significantly reduced even though it is not completely forbidden by symmetry, and we find a lower energy splitting between the hybrid MIDGAP-l and MIDGAP-h states (0.14 eV) in Fig. 3(c). The same electronic configuration discussed above can be realized by a 1s→LUMO excitation involving a minority spin core electron. In case a majority 1s state is excited into the valence shell, the spin of the added electron and of the mid gap state sums up leading to a system with 2 *μ*_*B*_ magnetic moment, eventually occupying singly both MIDGAP-l and MIDGAP-h with the same spin. Such a configuration is less stable than the non-magnetic case by 0.36 eV. Relaxation towards the spin-compensated solution could occur by resonant transfer of two electrons with majority and minority spin to and from the surrounding graphene, respectively, provided this is faster than the core-hole lifetime.

**Figure 5:**
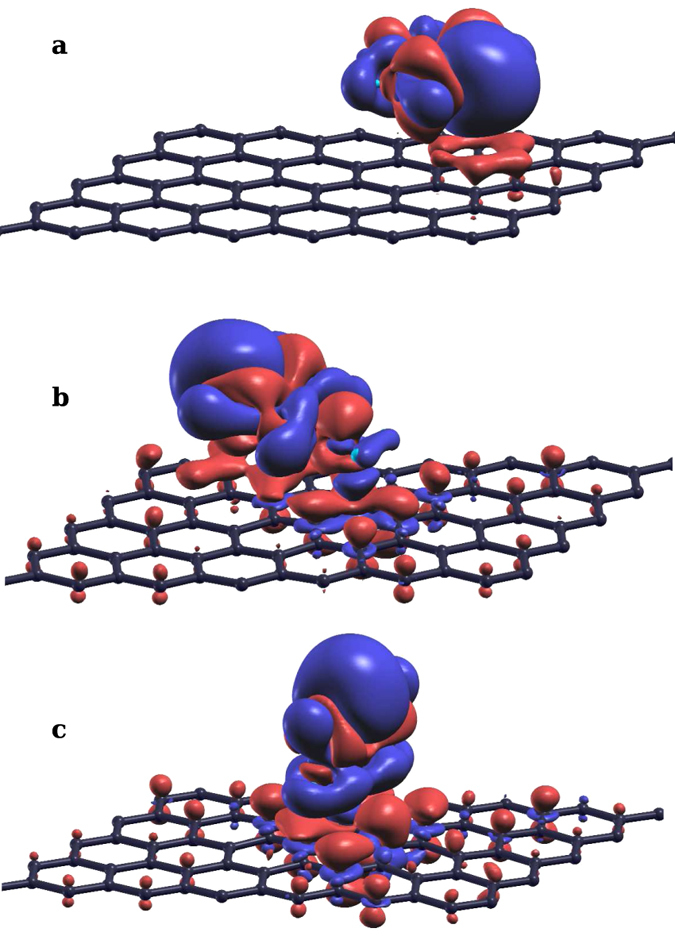
Screening charge for (**a**) pyridine, (**b**) 4-picoline radical and (**c**) pyridine radical. The isovalue is 0.0034 e/Å^3^ and regions of electron accumulation/depletion are depicted in red and blue regions, respectively.

A further distinction between the different adsorption systems can be inferred by calculating the screening charge which is defined as 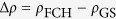 (here *ρ*_FCH_ and *ρ*_GS_ are electron density of the system in the core ionized and the ground state, respectively), whose integral amounts to the additional valence electron. Let us consider first the results for physisorbed pyridine shown in Fig. 5(a). Here, the screening is mostly concentrated in the molecular region where a polarization of the molecular orbitals towards the core hole as well as a filling of the LUMO, can be appreciated. By partitioning Δ*ρ* in terms of projection onto atomic orbitals according to Löwdin population analysis^65^,^66^, we found that 70% of the added charge can be attributed to the atoms of the molecule and the remaining 30% to graphene, in agreement with the occupation of the states seen in Fig. 3(a). In the case of the chemisorbed molecules, we have a more pronounced participation by the graphene substrate to the screening: one switches from the spin-polarized mid gap state occupied by one electron, to the spin-degenerate hybrid one. MIDGAP-l depicted in Fig. 4(a,c), is now doubly occupied. The participation of graphene to the screening charge is moderately larger for pyridine radical than for 4-picoline radical. This can be seen visually by comparing Δ*ρ* and noticing that larger charge lobes on graphene are depicted in Fig. 5(c) than in Fig. 5(b). Correspondingly, the projection of Δ*ρ* on the atoms of the molecule amounts to 63% for the 4-picoline radical and 59% for the pyridine radical cases.

Finally we remark that we deal with a local phenomenon independent of possible long range magnetic order. Given the limited extent of the core-level excitation, experiments to detect the local spin balance are conceivable when in the presence of the core hole, electrons are transferred from the substrate to the molecular LUMO level^45^. By measuring just above the relevant absorption edge, the spin state of low kinetic energy photoemitted electrons in coincidence with the spin resolved spectra of high energy electrons originated from the relaxation of electrons transferred to the LUMO state, a difference depending from the relative spin state should be detectable. These experiments are surely at the limit of today’s experimental techniques but are not unfeasible. We recall that photo- and Auger-electron coincidence measurements have already been used in the past to identify the local magnetic behavior in ferromagnetic^67^ and antiferromagnetic^68^ materials, exploiting the interplay between the relative electron spins and the angular dependence of the emitted electrons. In the case of a distribution of chemisorbed radicals, networks of spin chains could be obtained attaining long range magnetic order^33^. By the process described in this paper the destruction of magnetic correlation in the fs time domain could be induced by core excitation of a few adsorbates under X-ray irradiation.

## Conclusions

We have studied the magnetic properties of ground state and core-excited organic molecules on graphene from first principles. It is found that the adsorption mechanism plays an important role in inducing or suppressing magnetism in the valence shell of the system within the core hole lifetime. The physisorbed system where the interaction is dominated by van der Waals forces and initially non-magnetic in the ground state becomes magnetic when a core electron is excited. The system is magnetic until the core electron de-excites via one of the several electronic decay channels. An opposite behaviour can be appreciated when molecules are covalently bonded to graphene and the substrate is hence magnetic. Here magnetism is suppressed upon core electron excitation. The LUMO of the molecule hybridizes with the mid gap states of graphene forming spin-compensated bonding and anti bonding combinations. The valence shell remains non magnetic until the core electron de-excites. Since the time scale of the magnetism we are dealing with is in the order of a few femtoseconds and considering the recent advances in femtomagnetism and ultrafast measurements for adsorbed molecules, experiments complementing our work could soon be available.

## Additional Information

**How to cite this article**: Ravikumar, A. *et al*. Femtomagnetism in graphene induced by core level excitation of organic adsorbates. *Sci. Rep.* **6**, 24603; doi: 10.1038/srep24603 (2016).

## Supplementary Material

Supplementary Information

## Figures and Tables

**Table 1:** The adsorption energies (E_*ads*_), graphene-molecule bond length (*a*_*cc*_) and graphene carbon atom shift (*a*_*Z*_) are tabulated for the three species adsorbed on graphene.

**Configuration**	***E***_***ads***_ **(eV)**	***a***_***cc***_ **(Å)**	***a***_***Z***_ **(Å)**
Pyridine	−0.60	3.22	—
4-Picoline radical	−0.51	1.64	0.41
Pyridine radical	−0.22	1.58	0.54
