# Supplementary material for: Femtomagnetism in graphene induced by core level excitation of organic adsorbates
Source: Sci Rep. 2016 Apr 19;6:24603. doi: 10.1038/srep24603 (PMC4835731; doi:10.1038/srep24603)
Supplement: Supplementary Information [file srep24603-s1.pdf]

Supplementary Information for

Femtomagnetism in graphene induced by core level excitation of organic adsorbates

Abhilash Ravikumar[a], Anu Baby[a], He Lin[a], Gian Paolo Brivio[a], Guido Fratesi[b,a]

[a] *Dipartimento di Scienza dei Materiali, Università di Milano-Bicocca, Via Cozzi 55 - 20125 Milano, Italia. Fax:+39-02-644854000; Tel: +393451475855; E-mail:a.ravikumar@campus.unimib.it*

[b] *Dipartimento di Fisica, Università di Milano, Via Celoria, 16 - 20133 Milano, Italia; E-mail:guido.fratesi@unimi.it*

Evaluation of the molecular orbital projected density of states

We describe here the procedure to compute the Molecular Orbital Projected Density of States (MOPDOS) that we have recently implemented in the `molecularpdos.x` code within the Quantum ESPRESSO distribution [1]. As we used in the current framework, the aim is to single out the contributions to the density of states of an adsorbed system coming from the orbitals of the free molecule. In more general terms, one can analyze the electronic structure of a given system named "A" (here, the molecule/graphene interface) in terms of the energy levels of a part of it named "B" (here, the molecule). This approach may also be used to analyze a complex molecule in terms of its subunits, or a different electronic configuration (say, A is the molecule with a core-level excitation while B is the same but in the ground state as we did to study excitations of pentacene [2]).

If we indicate by $|\psi^A_{n_a\mathbf{k}}\rangle$ the eigenvectors of system A and by $\epsilon^A_{n_a\mathbf{k}}$ its eigenvalues (same for system B), where $\mathbf{k}$ is the k-vector in the Brillouin zone with weight $\omega_\mathbf{k}$, the MOPDOS of system A, projected onto the n_b-th orbital of system B and evaluated at the energy E reads:

$$MOPDOS_{n_b}(E) = \sum_{n_a\mathbf{k}} \omega_\mathbf{k}\ |\langle\psi^B_{n_b\mathbf{k}}|\psi^A_{n_a\mathbf{k}}\rangle|^2\ \delta(E - \epsilon^A_{n_a\mathbf{k}}). \tag{1}$$

The eigenstates are computed by separate `pw.x` calculations for A and B; for consistency, the same unit cell and $\mathbf{k}$ sampling should be used.

A shortcoming of the plane wave representation is that direct evaluation of the overlap integral $|\langle\psi^B_{n_b\mathbf{k}}|\psi^A_{n_a\mathbf{k}}\rangle|^2$ requires handling the full states for the two systems, which can be computationally demanding already for a moderately large unit cell (of the order of 10^6 plane waves or real space points in the case presented here, for each $\mathbf{k}$ and orbital). Our implementation instead goes through a more efficient local basis set representation of the system. Let us indicate by $|\phi^{nlm}_I\rangle \equiv |\phi_\nu\rangle$, with $\nu = (I, n, l, m)$, the atomic wavefunction of atom I with quantum numbers n, l, and m. The number of such states, N_ϕ, is generally much smaller than that of plane waves making the calculations more manageable (here, $N_\phi \approx 300$). Hence we can approximate the eigenfunctions in terms of this local basis set:

$$|\psi^A_{n_a\mathbf{k}}\rangle \approx \sum_\nu P^A_{n_a\mathbf{k},\nu}|\phi_\nu\rangle \tag{2}$$

$$|\psi^B_{n_b\mathbf{k}}\rangle \approx \sum_\nu P^B_{n_b\mathbf{k},\nu}|\phi_\nu\rangle \tag{3}$$

where the coefficients P are the complex projections of the Kohn-Sham eigenstates onto the local basis, $P^A_{n_a\mathbf{k},\nu} = \langle\phi_\nu|\psi^A_{n_a\mathbf{k}}\rangle$ and similarly for B. The above expressions are approximate since the local basis set does not span completely the original Hilbert space (see, e.g., the "spilling" [3]) but this is often of no concern to a qualitative analysis. Within Quantum ESPRESSO,

the coefficients P are computed by the `projwfc.x` code in a standard calculation of the DOS projected onto atomic orbitals and are stored in the file `atomic_proj.xml`. The execution of `projwfc.x` has to be performed for systems A and B separately.

The orbital overlaps in Eq. (1) are eventually computed from Eqs. (2) and (3) as:

$$\langle \psi^B_{n_b\mathbf{k}} | \psi^A_{n_a\mathbf{k}} \rangle = \sum_\nu (P^B_{n_b\mathbf{k},\nu})^* P^A_{n_a\mathbf{k},\nu}. \qquad (4)$$

Notice that the index ν in the summation should identify the same atomic state in the two systems for the local orbitals which are common for the two systems only. So, if system B is a subsystem of A, we have $N^A_\phi > N^B_\phi$ and the summation runs over N^B_ϕ states. We remark that, for an adsorbed radical, where the dangling bond is saturated by the surface, system B is most effectively taken as the radical saturated by an hydrogen atom whose atomic state should not be included in Eq. (4). In all cases, the fraction of atomic states to be used can be specified in input by appropriate variables (`i_atmwfc_beg_full/part` and `i_atmwfc_end_full/part`), as illustrateed by the following sample input for a pyridine radical adsorbed on 5×7 graphene:

```
&INPUTMOPDOS
  xmlfile_full='full/atomic_proj.xml'
  i_atmwfc_beg_full=1
  i_atmwfc_end_full=28
  xmlfile_part='part/atomic_proj.xml'
  i_atmwfc_beg_part=2
  i_atmwfc_end_part=29
  i_bnd_beg_part=15
  i_bnd_end_part=16
```

```
  ... plotting ranges
  ... broadening parameters
/
```

The full system (A) has 75 C atoms (70 from graphene and 5 from the molecule) and 1 N atom with 2s and 2p orbitals and 4 H atoms with 1s orbitals for a total of $N_\phi^A = 75 \times 4 + 1 \times 4 + 4 \times 1 = 308$, 280 from graphene and 28 from the radical. The saturated molecule (B) has one H atom more hence $N_\phi^B = 29$ atomic orbitals. In the above example we project all states of the full system onto the molecular HOMO and LUMO (orbitals 15 and 16), assuming that the graphene atoms are listed last in system A and that the H atom saturating the dangling bond (to be neglected) is given first in system B. Had we saturated the radical with a methyl group, 7 atomic orbitals should have been neglected from system B.

References

[1] P. Giannozzi, et al. "QUANTUM ESPRESSO: a modular and open-source software project for quantum simulations of materials." Journal of Physics: Condensed Matter 21.39 (2009): 395502.

[2] A. Baby, H. Lin, G. P. Brivio, L. Floreano, and G. Fratesi, "Core-level spectra and molecular deformation in adsorption: V-shaped pentacene on Al(001)" Beilstein J. Nanotechnol. 6, (2015): 2242-2251.

[3] D. Sanchez-Portal, E. Artacho, and J. M. Soler. "Projection of plane-wave calculations into atomic orbitals." Solid State Communications 95.10 (1995): 685-690.
